# Treatment of the Morphine Habit

**Published:** 1892-03

**Authors:** James Johnston

**Affiliations:** Brownwood


For Daniel's Texas Medical Journal.

### TREATMENT OF THE MORPHINE HABIT.

BY JAMES JOHNSTON, M. D., BROWNWOOD.

THE magnitude of the evil of the opium habit is creating some desire on the part of the medical profession, both in Europe and this country, to investigate this subject, and for the past year more attention has been paid to the scientific treatment of this evil than for the previous fifteen years.

It is not my intention to give a description of the debasing effect of the morphine habit on society, or a systematic amount of the pathology and symptoms of its course, but a brief outline of the treatment that has proved successful in my hands.

Medical treatment includes all that is done by the physician for the relief of the unfortunate victim, whether it belongs to the domain of allopathy, hydropathy, homeopathy, electropathy, massage, etc., and as I have heard Prof. Marco remark, "A little professional deception may be necessary." Although the treatment, under certain conditions properly carried out, is as successful as the treatment of any disease known to medical science, yet without strict attention to numerous details it becomes a very difficult matter. No treatment requires so many *small things* to be done, with perfect submission to all the requirements, on the the part of the patient, and special knowledge, experience and tact on the part of the physician. One of the principal conditions for successful treatment is a sincere desire on the part of the patient to be cured, a willingness to submit to all the regulations of a well ordered institntion and a co-operation with the physician.

The next necessary condition for a cure is freedom from chronic or functional diseases, which require the constant use of an opiate to relieve pain, such as malignent tumors, neuralgia, angina-pectoris, epilepsy, hysteria, and nervous diseases in general, etc.

Some of the above conditions, having been caused by the use of opiates, may disappear under the opium treatment.

Another important condition for successful treatment is perfect freedom from business and business cares, mental anxiety, family trouble, financial embarassment, etc. Professional men are hard to cure, because they will not give up entirely the pressing duties that usually devolve upon them.

Large public institutions have not been found favorable places for the successful treatment of morphine habitues. Persons addicted to strong drink are hard subjects, and cannot be successfully treated unless they give up the use of strong drink, or undergo treatment for both habits at the same time.

Climate has something to do with the treatment. Patients are easier cured in a warm or temperate climate than in a cold one. Morphine habitues will tell you that they require to take a larger amount of the accustomed drug in cold weather than in moderately warm weather.

The treatment I have found most satisfactory is the gradual reduction plan. After twenty-five years study of this subject, and several years of actual observation, I find that this is the only humane and practical treatment; notwithstanding such men as Levinstein, of Berlin, and some other European specialists, advocate sudden and complete deprivation of the drug, but this causes torture worse than death. To say the least, it is *barbarous in the extreme*, and according to statistics, very unsuccessful. Nine patients out of ten relapsed immediately after leaving Levinstein's institution.

When a patient is put under treatment he should be required to give up all opiates, and give a solemn promise that he is not to leave the premises without permission, or to use anything in the shape of drugs or medicines unless prescribed for him; he should be put on nerve tonics, and the amount of the accustomed drug reduced, in most cases, $\frac{1}{4}$; after four to six days, another reduction is made, say $\frac{1}{6}$, and next $\frac{1}{8}$, $\frac{1}{10}$, $\frac{1}{12}$, $\frac{1}{14}$, $\frac{1}{16}$, and so on, until the daily amount is reduced to less than two grains of morphine. Codea is then substituted. This article is found to produce almost the same effect as morphine, but as it is only one-fourth its strength, must be given in larger doses. It has the advantage of not creating a necessity for its continued use, therefore, it can be left off, and the dose does not require to be constantly increased to produce the effect as with morphine. Unless the reduction is exceedingly slow, and the nervous system well restored with nerve tonics, considerable disturbance of the system will take place at the final breaking off.

The most prominent danger is heart failure, and the peculiar condition of the pulse indicating it cannot be well determined without the aid of the sphygmograph; with this ingenious delicately constructed instrument the platen of the pulse can be accurately determined;--the heart must be sustained, and this is accomplished by the use of heart tonics. Their efficacy will be found in the following order: Trinatein, nitrite of amyl, spartenia and digitalis. Most of these can be used hypodermically. The objection to the general administration of the first two is that they produce a severe headache in many cases. When this occurs spartenia and digitalis must be used. Spartenia has the advantage of an eliminator, on account of its diuretic properties. I am indebted to Orcar Jennings, M. D., of Berlin, for valuable suggestions on the use of the sphygmograph and the effacacy of heart tonics towards the end of the treatment, and I find very flattering results from the practical application of his theory with some recent cases under my care.

Restlessness and pains in various parts of the body must be relieved by baths and electricity. The hot air and Turkish baths are the best for this purpose. Every organ must be spared unnecessary strain. Exercise must be very moderate. The liver is usually enlarged, and sluggish; gastralgia may be relieved by a blister over the region of the stomach and the free use of flaxseed tea. Alkiline solutions increase the strength of the heart's action, and cause contraction of the capillaries. Bromide of potassium calms the excitement of the heart and cerebral centers, more especially when it is combined with spartenia or digitalis. Sulphonal and valerianate of ammonia and cannabis indica are said to be useful, but I have not sufficient experience with them to speak of their effects. Coca may be used to some advantage at the close of the treatment for a short time, but its physiological effects are so very uncertain that it should not be used except in the hands of a specialist of extensive experience; a habit for its use is easily formed. The same may be said of hydrate of chloral.

Two courses of treatment are open to patients; treatment at home, or treatment in a special establishment. The latter has many advantages; many cases can be cured at a well conducted sanitarium, which would fail with the home treatment. In either case, patients must leave off the use of opiates in any form, and submit themselves to be treated as if suffering from any other disease; and the same degree of secrecy with regard to the nature of the medicine prescribed must be observed. If patients are aware of the fact that they are taking morphine in regular diminished doses they are very likely to indulge in taking an extra dose or using morphine (if they can obtain it) in addition to the medicine prescribed, and failure is the result. Patients will submit to, and are more reasonable with strangers than with members of their own family, and will adopt and carry out treatment prescribed by a physician at a distance in preference to that prescribed by their family physician.

The careless, easy, indifferent manner of the opium habitue must be overcome by strict discipline, well carried out, and this cannot be accomplished, except at some private institution. Physical restraint is wrong, but the patient should submit to restriction.

There is much difference in the condition of various patients seeking treatment, and also in the quantity of opiate used daily. Some have been successfully cured who were in the habit of taking seventy-five grains or morphine per day, where other cases have failed, or relapsed, where eight grains per day was the dose. More depends on the physical and mental condition of the patient than on the quantity of the drug taken. All the organs of the body should be kept in good order; light nutrious diet without stimulants is necessary, and moderate exercise without fatigue is also beneficial. Patients treated at their own homes require a much longer time to get free from the habit, as they are not under the supervision of the physician, and if subjected to suffering, there is no one at hand to relieve them, and the consequence is a return to the accustomed drug.

I am aware that morphine habitues are hard to treat in private practice, but I think a sufficient number can be cured at their own homes by their family physician to make this important subject a matter of investigation and study. Sufferers naturally look to the regular profession for relief, and when they fail, become the prey of unscientific persons, whose only object is pay. This subject is worthy of our best thought and endeavors.

